# Assessment of Tribological Properties of Ti_3_C_2_ as a Water-Based Lubricant Additive

**DOI:** 10.3390/ma13235545

**Published:** 2020-12-04

**Authors:** Huong Thi Nguyen, Koo-Hyun Chung

**Affiliations:** School of Mechanical Engineering, University of Ulsan, Ulsan 44610, Korea; mailanhuong95@gmail.com

**Keywords:** titanium carbide, friction, wear, water lubrication, lubricant additive

## Abstract

Water-based lubrication has attracted remarkable interest due to its environmental and economic advantages. However, practical applications of water-based lubrication are often limited, mainly because of low viscosity and corrosivity. The use of additives has been proposed to overcome these limitations. In this work, the tribological characteristics of titanium carbide (Ti_3_C_2_) MXenes, as additives for water-based lubrication, were systematically investigated for contact sliding between stainless steel under various normal forces and Ti_3_C_2_ concentrations. Both friction and wear were found to decrease with increasing Ti_3_C_2_ concentration up to 5 wt%, and then increased when the concentration was larger than 5 wt%. The results suggest that Ti_3_C_2_ flakes hindered direct contact, particularly at the edges of the contact interfaces. It was further shown that the agglomeration of Ti_3_C_2_ flakes may have reduced the hindering when an excessive amount of Ti_3_C_2_ (e.g., 7 wt%) was applied. The decreases in the friction coefficient and wear rate with 5 wt% of Ti_3_C_2_ concentration w approximately 20% and 48%, respectively. The outcomes of this work may be helpful in gaining a better understanding of the tribological properties of Ti_3_C_2_ as a feasible water-based lubrication additive.

## 1. Introduction

Environmental degradation, such as resource depletion, climate change, and pollution, has become a growing concern in the last few decades. To overcome these problems, and to ensure a sustainable future, various green technologies have been under development [1,2,3]. In particular, green tribology involves the minimization of friction and wear, environment-friendly or biodegradable lubrication, reduction or even elimination of lubrication, complete utilization of materials, biomimetic surface design, and tribology for renewable sources of energy, with the aim of saving energy and materials, and minimizing the harmful impacts on the environment and ecological balance [4,5,6]. Among the green tribology solutions currently under development, water-based lubrication technology, which potentially can replace conventional oil-based lubricants, has attracted considerable interest due to its economic and environmental benefits, such as low cost, cleaning performance, natural resource conservation, and environmental sustainability.

Water-based lubrication is often limited in practical tribological applications, mainly due to its low viscosity and corrosivity. In order to overcome these limitations and improve the properties of water-based lubricants, extensive efforts have been made over the past decades. For example, fundamental studies to understand the performance of water-lubricated journal bearings have been performed [7,8]. The tribological properties of various coatings with good lubricity in a water environment have also been investigated [9,10]. In addition, to enhance the tribological properties of the coatings in water-based lubrication, surface texturing, such as nanostructure, micro-dimples, and grooves, has been proposed [11,12,13]. Furthermore, investigations have been conducted on the use of additives to overcome the limitations of water-based lubrication [14]. Various nanoparticles, including copper (Cu) [15], diamond [16], titanium dioxide (TiO_2_) [17], and silicon dioxide (SiO_2_) [18], have been proposed as potential candidates for lubricant additives. Carbon-based nanomaterials, such as fullerenes [19] and carbon nanotubes [20], have also attracted considerable interest as additives for water-based lubrication. Particularly, graphene and its derivatives have recently been demonstrated as promising lubricant additives, due to their low friction characteristics, associated with weak interatomic interactions between layers, and chemical inertness [21,22,23]. For example, when graphene oxide was added to water, it was observed that the friction coefficient was maintained at approximately 0.05 for up to 60,000 cycles, without any significant wear [22]. It was demonstrated that the decrease in friction and wear may have been associated with the formation of a tribo-film that can act as a protective coating [22,23]. Furthermore, considering that other two-dimensional (2D) layered materials, such as molybdenum disulfide (MoS_2_) and hexagonal-boron nitride (h-BN), can provide low-frictional properties [24,25,26], their tribological characteristics as additives for water-based lubrication have been explored [27,28]. 

Recently, 2D layered transition metal carbides and nitrides, MXenes, have attracted attention as candidates for solid lubricants [29,30]. The general structure of MXenes is M_n+1_X_n_T_x_, where M is an early transition metal, X is C and/or N, and T represents a functional group. Titanium carbide (Ti_3_C_2_), often denoted as Ti_3_C_2_T_x_, where T is F, OH, and O, is one of the most extensively studied MXenes due to its potential for various applications [29]. It was shown that Ti_3_C_2_ may provide a significant reduction of friction and wear, attributed to the prevention of direct contact and the formation of a carbon-rich tribo-film at the contact interface [31]. The tribological properties of Ti_3_C_2_ were further correlated with surface terminations and intercalated water [32]. In particular, a practical approach for the use of Ti_3_C_2_ as a solid lubricant for thrust ball bearings was conducted [33]. Furthermore, it was demonstrated that Ti_3_C_2_ can be exploited as a lubricant additive to oil- and water-based lubricants [34,35,36,37,38]. However, more data should be accumulated on utilizing Ti_3_C_2_ as a lubricant additive. 

In this work, the tribological properties of Ti_3_C_2_ as an additive in water-based lubrication were experimentally investigated using a ball-on-disk tribotester. Experiments were performed using a stainless steel (SS) ball and disk at boundary lubrication, under various normal forces and Ti_3_C_2_ concentrations in water. The variation in friction was monitored during the tests and the wear rates of the specimens were quantitatively assessed using a laser scanning confocal microscope (LSCM) after the experiments. A better understanding of the wear behavior with Ti_3_C_2_ additives was additionally pursued using scanning electron microscope (SEM) observations. The outcomes may provide useful information to understand the effect of Ti_3_C_2_ on the tribological characteristic of SS under water-based lubrication. 

## 2. Materials and Methods

### 2.1. Materials

**Figure 1:**
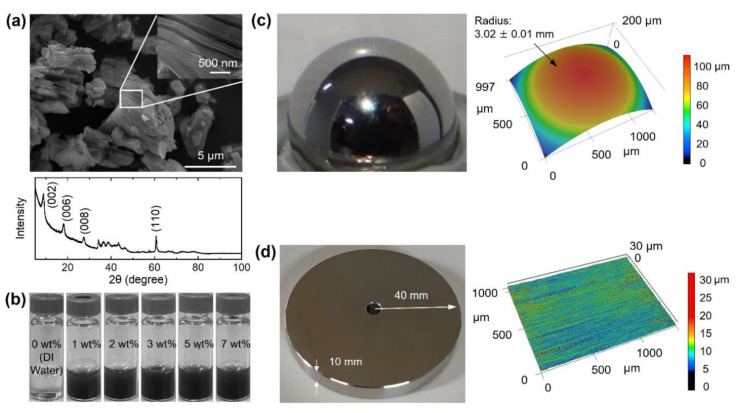
(**a**) SEM images and XRD pattern of Ti_3_C_2_ flakes, (**b**) photographs of water-based lubricants with 0 wt%, 1 wt%, 2 wt%, 3 wt%, 5 wt%, and 7 wt% Ti_3_C_2_ concentrations, and photographs and 3D laser scanning confocal microscope (LSCM) images of the (**c**) ball and (**d**) disk. Inset in (**a**) is a high-magnification of the SEM image.

Commercialized Ti_3_C_2_ flakes with 99.9% purity (Wuhan Golden Wing Industry & Trade, Wuhan, China) were used in this work. The nominal size of the flakes was 0.2–3 μm. Figure 1a shows SEM images of Ti_3_C_2_ flakes. It can be seen from the figure that the lateral size of Ti_3_C_2_ flakes varied from less than 1 μm to several μm. The high-magnification SEM image inset in Figure 1a shows clearly the layered structure of Ti_3_C_2_. In addition, the thickness of the layer was found to be approximately 20 nm, which was consistent with the thickness reported in the literature [34,37]. The X-ray diffraction (XRD) pattern of the flakes is also provided in Figure 1a, indicating the formation of Ti_3_C_2_ [39]. Five solutions with different concentrations of Ti_3_C_2_ in pure deionized (DI) water were prepared, with the aim of investigating the effect of concentration on friction and wear characteristics. To prepare the solutions, 0.01 g, 0.02 g, 0.03 g, 0.05 g, and 0.07 g of Ti_3_C_2_ were added to 1 mL of DI water for the solutions with 1 wt%, 2 wt%, 3 wt%, 5 wt%, and 7 wt% Ti_3_C_2_ concentrations, respectively. Then, each solution was stirred in a magnetic stirrer for 1 h at room temperature to ensure that the Ti_3_C_2_ was uniformly dispersed. Figure 1b shows photographs of the DI water and the DI water solutions with five different concentrations of Ti_3_C_2_ additives.

Martensitic SS (AISI 440C) was selected as the ball material due to its high hardness and corrosion resistance in water. The nominal radius of the balls was 3 mm. All balls used in this work were carefully examined under a LSCM (VK-X200, Keyence, Osaka, Japan) to determine the radii, and to assess their surfaces before experiment. Figure 1c shows a photograph of the ball glued on the holder for the experiment, along with an example of a three-dimensional (3D) LSCM image before experiment. The average radius of the balls was calculated to be 3.02 ± 0.01 mm (mean ± one standard deviation). It can be seen from Figure 1c that the surface of the ball before experiment was quite clean. The average surface roughness (*R_a_*) of the balls was also determined from the LSCM data with a scan size of 1270 µm × 997 µm after the flattening process. The *R_a_* value of the balls was calculated to be 0.29 ± 0.01 µm. The hardness of the balls was further determined using a micro-Vickers hardness tester (MMT-7, Matsuzawa, Japan). To that end, a ball was ground to form a flat surface. Hardness measurement was performed with an indentation force of 0.98 N and dwell time of 20 s at five different randomly selected locations on three balls. The hardness values of the three balls were found to be similar to one another (765 ± 28 HV, 785 ± 34 HV, and 730 ± 10 HV). The average hardness value was determined to be 760 ± 23 HV. The hardness values of the balls determined in this work were in good agreement with those of AISI 440C SS.

Austenitic SS (AISI 304) was used as the disk material. SS disks with a radius of 40 mm and a thickness of 10 mm were prepared for the experiment. Figure 1d shows the photograph and 3D LSCM image of a disk before experiment. From Figure 1d, the patterns, which likely formed during the manufacturing process, can be observed. The *R_a_* value of the disks was determined to be 1.67 ± 0.05 µm from the LSCM data, with a scan size of 1408 µm × 1056 µm obtained at five different locations on five different disks. The hardness of the disks was measured from five different locations of five disks using the micro-Vickers hardness tester (MMT-7, Matsuzawa, Japan), with an indentation force of 0.98 N and dwell time of 20 s. The average hardness value of the disks was 220 ± 10 HV, which was within the typical range of hardness of AISI 304 SS. 

### 2.2. Methods

**Figure 2:**
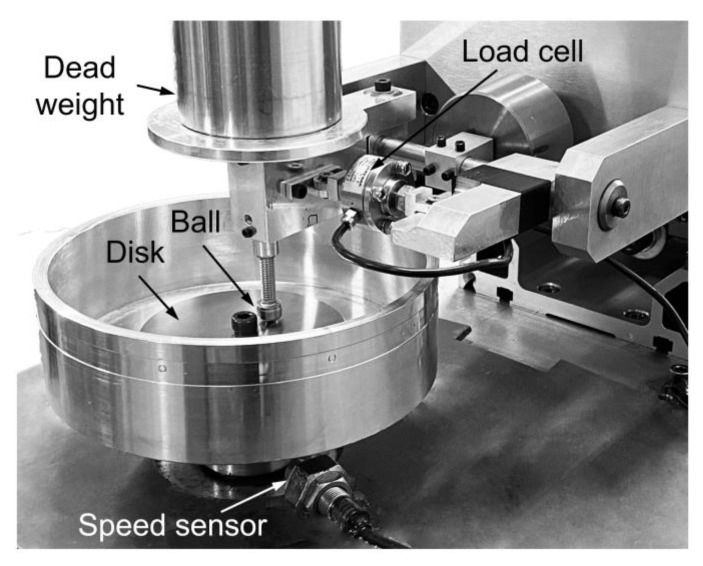
Photograph of the ball-on-disk tribotester used in this work.

The tribological properties of Ti_3_C_2_ as a water-based lubricant additive for contact sliding between a SS ball and a disk were investigated using a ball-on-disk tribotester under boundary lubrication. A photograph of the tester used in this work is shown in Figure 2. A normal force was applied by a dead weight, and the friction force was monitored using a load cell equipped with the tester. The experiments were conducted for a specimen radius of 10 mm, therefore the sliding distance of 1 cycle was 0.063 m. The rotating speed was set to 120 rpm, which corresponds to a linear sliding speed of 0.126 m/s. The normal force varied from 3 N to 10 N, which corresponded to a contact pressure from 0.91 to 1.36 GPa, as calculated using Hertzian contact model [40]. The experiment with a given normal force was conducted for up to 7000 cycles. A 0.2 mL quantity of lubricant was applied to the contact area using a syringe immediately after preparation, to minimize the effect of dispersibility of additives on tribological performance. The experiments were repeated at least three times for each experimental condition. Prior to the experiments, the balls and disks were cleaned with isopropyl alcohol (IPA) by ultrasonication for 1 h. All tests were performed in ambient conditions with the temperature 24 °C–26 °C and relative humidity 37–46%.

The surface of the balls and the wear track formed on the disks were carefully examined after experiments using the LSCM to understand the wear characteristics of water-based lubricants with Ti_3_C_2_ additives. In particular, the wear rates of the ball and disk were carefully determined using LSCM data. To that end, additional LSCM images of the balls and disks were acquired after ultrasonic cleaning, since a significant amount of wear debris and additives may have been readily deposited on the surfaces during the experiment. The wear volume of the balls was determined as the volume of the spherical segment calculated using the cross-sectional height profiles at the center of the ball before and after experiment, considering that the ball was flattened due to wear. As for the disks, LSCM images were obtained at five different locations of the wear track, and averaged cross-sectional height profiles were taken from each image. Then, the wear volume of the disk was calculated by multiplying the average cross-sectional area of the wear track by the circumferential contact length. Furthermore, SEM observations and energy-dispersive X-ray spectroscopy (EDS) analyses were performed, particularly of the Ti_3_C_2_ flakes after the experiment, to gain a better understanding of tribological behavior of water-based lubricants with Ti_3_C_2_ additives. 

## 3. Results and Discussion

### 3.1. Friction Characteristics 

**Figure 3:**
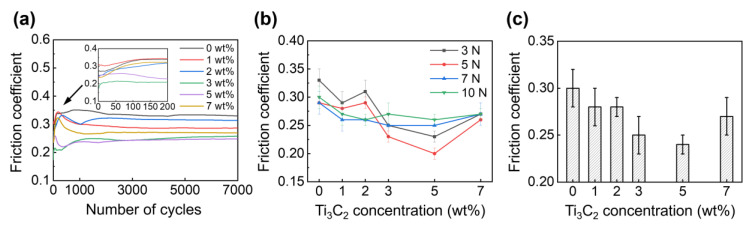
(**a**) Variation of friction coefficient with the number of cycles under 3 N normal force, (**b**) variation of friction coefficient at steady state with Ti_3_C_2_ concentration under 3 N–10 N normal force, and (**c**) variation of average friction coefficient with Ti_3_C_2_ concentration. Inset in (**a**) is the plot of friction coefficient for the number of cycles below 200.

Figure 3a shows the variation of friction coefficient with the number of cycles under a 3 N normal force, obtained using the water lubricants with 0 wt%, 1 wt%, 2 wt%, 3 wt%, 5 wt%, and 7 wt% Ti_3_C_2_ concentrations. The initial friction coefficient was found to range from 0.17 to 0.31. Additionally, it was shown that the friction coefficient fluctuated during the initial stage of contact sliding for up to 3000 cycles. Then, the friction coefficient became relatively stable, and ranged from 0.24 to 0.34, as the Ti_3_C_2_ concentration varied from 0 wt% to 7 wt%. It is plausible that the friction coefficient varies during the run-in process, and becomes stable with increasing the number of cycles [41]. A difference in the variation of friction coefficient during the run-in period could be observed, which was likely associated with the initial states of ball and disk surfaces, and Ti_3_C_2_ flakes at the contact interface. The difference in these initial states might have further led to the difference in duration of the run-in period. The data presented in Figure 3a show that the friction coefficients for the water lubricants with Ti_3_C_2_ additives were consistently lower than that without Ti_3_C_2_ additives. It can also be seen from the figure that the decrease in friction coefficient was the largest when the Ti_3_C_2_ concentration was 5 wt%. 

The friction coefficients were averaged after they became stable (i.e., from 3000 to 7000 cycles) for a clear comparison, and the variation of average friction coefficient was plotted against the Ti_3_C_2_ concentration under various normal forces, as shown in Figure 3b. It can be observed from the figure that the friction coefficient generally decreased as the Ti_3_C_2_ concentration increased up to 5 wt%, but it increased when the Ti_3_C_2_ concentration was 7 wt%, as anticipated from the data in Figure 3a. However, the effect of normal force on friction coefficient was not clearly observed. The friction coefficients for 0 wt% and 5 wt% Ti_3_C_2_ concentrations were found to range from 0.29 to 0.33, and from 0.20 to 0.26, respectively. The decrease in the friction coefficient for a 5 wt% Ti_3_C_2_ concentration was calculated to be approximately 13–31%, compared to that for a 0 wt% Ti_3_C_2_ concentration.

The friction coefficients obtained for normal forces ranging from 3 N to 10 N were further averaged to investigate more carefully the effect of Ti_3_C_2_ concentration, as shown in Figure 3c. It was found that the friction coefficients decreased by 7%, 7%, 17%, 20%, and 10%, for 1 wt%, 2 wt%, 3 wt%, 5 wt%, and 7 wt% Ti_3_C_2_ concentrations, respectively, compared to the friction coefficient for a 0 wt% Ti_3_C_2_ concentration. These observations clearly suggest that Ti_3_C_2_ can reduce friction between a SS ball and a SS disk as an additive for water-based lubrication. The decrease in friction may be associated with behavior of the Ti_3_C_2_ (e.g., adherence to surface and trapping in grooves) that enters into the contacting interface, as proposed in previous studies [34,35,36,37]. Furthermore, it can be concluded that the optimal concentration of Ti_3_C_2_ for friction reduction was 5 wt%, for the considered material pair and experimental conditions adopted for this work. The friction reduction effect may likely decrease when an excess Ti_3_C_2_ is applied to the contact interface (e.g., Ti_3_C_2_ concentrations larger than 5 wt%), as consistently observed in previous investigations [34,35,36,37]. The degree of friction reduction by Ti_3_C_2_ for water-based lubrication was relatively smaller than that offered by graphene-based materials [22,23]. However, the decrease in the friction provided by Ti_3_C_2_ was comparable with those offered by other 2D materials, such as h-BN [27] and MoS_2_ [28], in water-based lubrication. Interestingly, the friction reduction obtained with Ti_3_C_2_ for oil-based lubrication [34,35,36,37] is generally smaller than when using graphene-based materials [42,43], but comparable to those offered by h-BN [44] and MoS_2_ [45]. It should further be noted that the degree of friction reduction by additives may significantly vary depending on experimental conditions. Nevertheless, the overall results in Figure 3 clearly suggest the feasibility of using Ti_3_C_2_ as an additive for water-based lubrication. 

### 3.2. Wear Characteristics 

**Figure 4:**
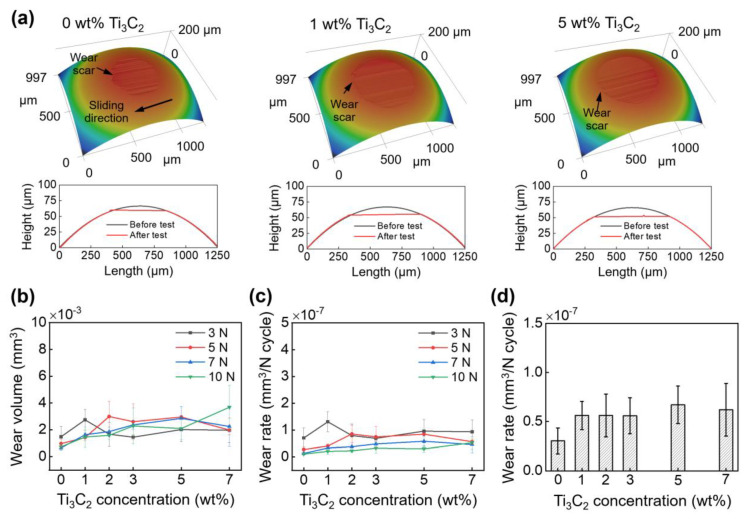
(**a**) 3D LSCM images and cross-sectional height profiles of balls after experiments with 0 wt%, 1 wt%, and 5 wt% Ti_3_C_2_ concentrations under 3 N normal force, (**b**) variation of ball wear volume and (**c**) wear rate under 3 N–10 N normal force with Ti_3_C_2_ concentration, and (**d**) variation of average wear rate with Ti_3_C_2_ concentration. In (**a**), cross-sectional profiles before experiments are included for comparison.

The wear characteristics of the balls and disks were assessed to elucidate further the feasibility of Ti_3_C_2_ as a water-based lubrication additive. Figure 4a shows 3D LSCM images of the balls cleaned using IPA after the experiments under a 3 N normal force. As examples, images of the balls tested using the water lubricants with 0 wt%, 1 wt%, and 5 wt% Ti_3_C_2_ concentrations are shown in Figure 4a. In Figure 4a, the cross-sectional height profiles before and after the experiment are provided for clear observation of wear. It can be seen from the figure that the balls were generally flattened due to the wear. In addition, it was observed that slight scratches appeared on the ball surface along the sliding direction. 

The ball wear volumes and wear rates for various normal forces are plotted as functions of Ti_3_C_2_ concentration in Figure 4b,c, respectively. The ball wear volume ranged from (6.4 ± 1.6) × 10^−4^ mm^3^ to (3.7 ± 1.6) × 10^−3^ mm^3^ under 3 N–10 N normal forces with 0 wt%–7 wt% Ti_3_C_2_ concentrations. No clear effect of the normal force on the ball wear volume was observed in Figure 4b. Additionally, it was shown that the effect of Ti_3_C_2_ concentration on the wear volume was not significant under a given normal force. The ball wear rate was calculated to be from (1.0 ± 0.3) × 10^−8^ mm^3^/(N·cycle) to (13 ± 3.7) × 10^−8^ mm^3^/(N·cycle) under 3 N–10 N normal forces with 0 wt%–7 wt% Ti_3_C_2_ concentrations, as shown in Figure 4c. As predicted from the data shown in Figure 4b, no significant dependence of the wear rate on the normal force and Ti_3_C_2_ concentration was further observed. This result suggests that the ball wear did not progress enough for the effects of normal force and Ti_3_C_2_ concentration to be observed clearly. For clarity, the variation of average ball wear rate with Ti_3_C_2_ concentration is plotted in Figure 4d. The ball wear rates with 1 wt%–7 wt% Ti_3_C_2_ concentrations were slightly higher than with 0 wt% Ti_3_C_2_ concentration, within the experimental uncertainties. As expected, the effect of Ti_3_C_2_ concentration on the ball wear rate was not significant. This result might be feasibly associated with the higher hardness of the ball than that of the disk, which resulted in the suppression of ball wear. A long-term test would be preferable for clear observation of the effects of normal force and Ti_3_C_2_ concentration on the ball wear.

**Figure 5:**
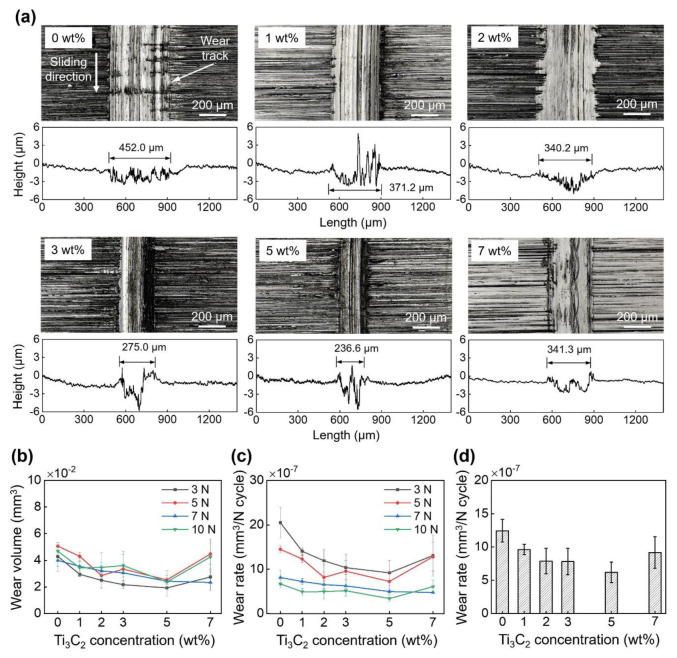
(**a**) LSCM images and cross-sectional height profiles of disks after experiments with 0 wt%, 1 wt%, 2 wt%, 3 wt%, 5 wt%, and 7 wt% Ti_3_C_2_ concentrations under 3 N normal force, (**b**) variation of disk wear volume and (**c**) wear rate under 3 N–10 N normal force with Ti_3_C_2_ concentration, and (**d**) variation of average wear rate with Ti_3_C_2_ concentration.

Figure 5a shows the LSCM images of the wear tracks formed on the disks after the experiments under a 3 N normal force for varying Ti_3_C_2_ concentrations. The averaged cross-sectional height profiles of the wear tracks are also presented in Figure 5a. The data in Figure 5a were obtained after the IPA cleaning. The images in Figure 5a demonstrate clearly that a significant amount of scratches were formed along the sliding direction on all disks. It was observed from the data in Figure 5a that in general, the effect of Ti_3_C_2_ concentration on the depth of the wear track was not consistent (depths of 1.64 µm, 0.6 µm, 1.49 µm, 0.52 µm, 0.50 µm, and 0.68 µm, for 0 wt%, 1 wt%, 2 wt%, 3 wt%, 5 wt%, and 7 wt% Ti_3_C_2_ concentrations, respectively). However, it can clearly be seen that the width of the wear track significantly decreased as the Ti_3_C_2_ concentration increased up to 5 wt% (widths of 452.0 µm, 371.2 µm, 340.2 µm, 275.0 µm, and 236.6 µm, for 0 wt%, 1 wt%, 2 wt%, 3 wt%, and 5 wt% Ti_3_C_2_ concentrations, respectively). Subsequently, the width of the wear track was found to increase (341.3 µm) when the Ti_3_C_2_ concentration increased to 7 wt%. The width of the wear track was expected to increase as the flattened area of the ball increased. However, no significant relationship between the flattened width of the ball and the width of the wear track on disk was observed from Figure 4a and Figure 5a. For example, although the flattened width of the ball after the experiment with a 7 wt% Ti_3_C_2_ concentration was larger than that after the experiment with 0 wt% Ti_3_C_2_ (Figure 4a), the wear track was wider for 0 wt% Ti_3_C_2_ concentration than for 7 wt% Ti_3_C_2_ concentration (Figure 5a). These results indicate that the direct contact between the ball and disk may have been hindered by the Ti_3_C_2_ flakes, particularly at the edges of the contact interface, and that such hindering by Ti_3_C_2_ flakes was more substantial as Ti_3_C_2_ concentration increased up to 5 wt%. However, it was postulated that this behavior of Ti_3_C_2_ flakes was reduced when an excessive amount of Ti_3_C_2_ flakes (e.g., 7 wt%) was applied to the contact interface.

The variation of disk wear volume with Ti_3_C_2_ concentration is presented in Figure 5b. The wear volume of the disks ranged from (1.9 ± 0.6) × 10^−2^ mm^3^ to (5.1 ± 0.3) × 10^−2^ mm^3^ as the normal force varied from 3 N to 10 N and the Ti_3_C_2_ concentration from 0 wt% to 7 wt%. Similarly to the wear of the balls, the effect of normal force on the disk wear volume was not clearly observed. However, it can be seen that the disk wear volume generally decreased as the Ti_3_C_2_ concentration increased to 5 wt%, and then increased when Ti_3_C_2_ concentration was 7 wt%, as expected from the data presented in Figure 5a. The decrease in the wear volume for 5 wt% Ti_3_C_2_ concentration (1.9 × 10^−2^ mm^3^) was as high as 55% under 3 N of normal force, compared to the wear volume for 0 wt% Ti_3_C_2_ concentration (4.3 × 10^−2^ mm^3^). 

Figure 5c shows the variation of disk wear rate for a given normal force with Ti_3_C_2_ concentration. It can be seen in the figure that the wear rate decreased with increasing normal force. For example, as the normal force increased from 3 N to 10 N, the wear rates for 0 wt% and 5 wt% Ti_3_C_2_ concentrations decreased from (20 ± 3.5) × 10^−7^ mm^3^/(N·cycle) to (6.7 ± 0.9) × 10^−7^ mm^3^/(N·cycle), and from (9.2 ± 2.9) × 10^−7^ mm^3^/(N·cycle) to (3.4 ± 0.7) × 10^−7^ mm^3^/(N·cycle), respectively. The Ti_3_C_2_ flakes may have been more damaged due to interactions with the ball, disk, and other Ti_3_C_2_ flakes under higher normal force. However, this may contribute to enhanced formation of a tribo-film that can suppress wear progression [34,35,36,37], which could be one of the reasons for the low wear rate under high normal force. In general, the wear rate was found to decrease with increasing Ti_3_C_2_ concentration from 0 wt% to 5 wt%, and then increase when Ti_3_C_2_ concentration was higher than 5 wt%. To clearly examine the effect of Ti_3_C_2_ concentration on the disk wear rate, the average wear rates for a given concentration are shown in Figure 5d. The wear rates were calculated to be (12 ± 1.7) × 10^−7^ mm^3^/(N·cycle), (9.6 ± 0.8) × 10^−7^ mm^3^/(N·cycle), (7.9 ± 1.9) × 10^−7^ mm^3^/(N·cycle), (7.8 ± 2.0) × 10^−7^ mm^3^/(N·cycle), (6.2 ± 1.5) × 10^−7^ mm^3^/(N·cycle), and (9.2 ± 2.4) × 10^−7^ mm^3^/(N·cycle) for Ti_3_C_2_ concentrations of 0 wt%, 1 wt%, 2 wt%, 3 wt%, 5 wt%, and 7 wt%, respectively. The decrease in wear rates for 1 wt%, 2 wt%, 3 wt%, 5 wt%, and 7 wt% Ti_3_C_2_ concentrations was calculated to be 20%, 34%, 35%, 48%, and 23%, respectively, compared to the wear rate for 0 wt% Ti_3_C_2_ concentration. The decrease in the disk wear rate for 5 wt% Ti_3_C_2_ concentration was relatively large, compared to that provided by h-BN [27], while it was relatively small, compared to that offered by graphene-based materials [22,23] for water-based lubrication. It is interesting to note that for oil-based lubrication the decrease in the wear rate provided by Ti_3_C_2_ [34,37] is often smaller than that provided by other 2D materials, including the graphene-based materials, h-BN and MoS_2_ [42,43,44,45]. Nonetheless, the overall results presented in Figure 5, along with the data in Figure 3, demonstrate the feasibility of Ti_3_C_2_ as a water-based lubrication additive for wear reduction.

**Figure 6:**
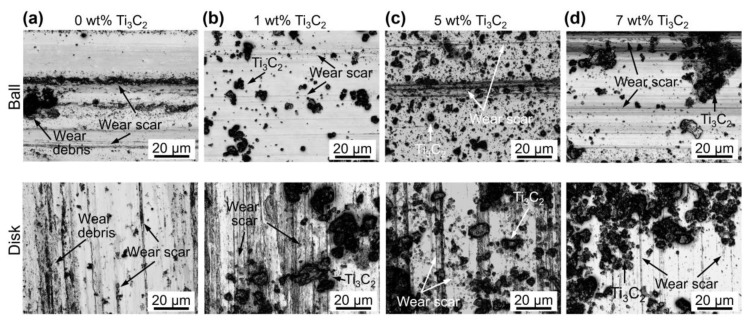
LSCM images of the flattened area of the ball and the wear track of disk after experiments with (**a**) 0 wt%, (**b**) 1 wt%, (**c**) 5 wt%, and (**d**) 7 wt% Ti_3_C_2_ concentrations under 3 N normal force.

To gain a better understanding of the effect of Ti_3_C_2_ on wear, the balls and disks were carefully examined before they were cleaned. As examples, the high-magnification LSCM images obtained after experiments with 0 wt%, 1 wt%, 5 wt%, and 7 wt% Ti_3_C_2_ concentrations are shown in Figure 6a–d, respectively. From the images in Figure 6a, along with those in Figure 4 and Figure 5, signs of abrasive and adhesive wear can be observed on both the ball and disk surfaces after the experiment with 0 wt% Ti_3_C_2_ concentration. However, the disk wear was more severe than the ball wear, which was due to the fact that the hardness of the disk was significantly smaller than that of the ball. In addition, a slight occurrence of abrasive wear was observed on the ball, which may be associated with the hard wear particles at the contact interface [46]. Figure 6a also shows the wear debris adhered to both the ball and disk surfaces. It was likely that the wear debris on the ball and disk surfaces was mainly generated from the disk, considering the disk wear volume was one or two orders of magnitude larger than the ball wear volume. 

**Figure 7:**
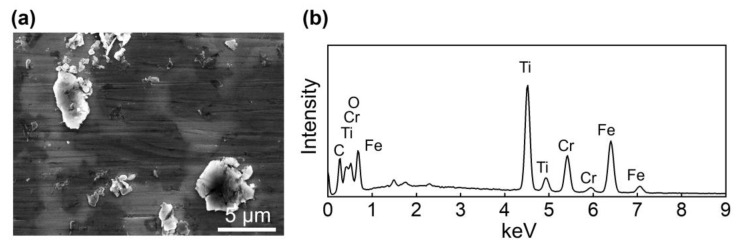
Examples of (**a**) SEM image of Ti_3_C_2_ and (**b**) EDS spectrum of Ti_3_C_2_ on the flattened area of the ball.

**Figure 8:**
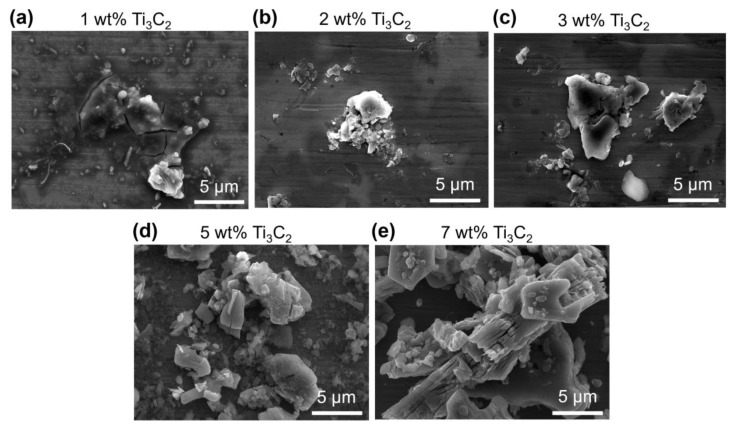
SEM images of the agglomerated Ti_3_C_2_ on the flattened area of the ball after experiments with (**a**) 1 wt%, (**b**) 2 wt%, (**c**) 3 wt%, (**d**) 5 wt%, and (**e**) 7 wt% Ti_3_C_2_ concentrations under 10 N normal force.

**Figure 9:**
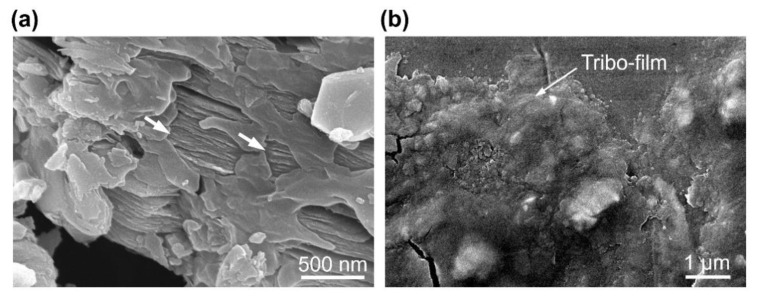
SEM images of (**a**) Ti_3_C_2_ and (**b**) tribo-film on the flattened area of the ball surface after experiment with 7 wt% Ti_3_C_2_ concentrations under 10 N normal force.

Figure 6 shows that the amount of particles on both the ball and disk surfaces generally increased with increasing Ti_3_C_2_ concentration. These particles were expected to be mostly Ti_3_C_2_ given that the wear volume of the ball and disk was a few orders of magnitude smaller than the volume of Ti_3_C_2_ in the lubricant. An SEM image and EDS spectrum of these particles on the ball surface are provided in Figure 7. The planar shape of the particles and the relatively strong Ti peak clearly indicate that these particles were mainly Ti_3_C_2_, as expected. The planar shape of Ti_3_C_2_ may be advantageous for it to enter the contact interface. It is likely that the Ti_3_C_2_ flakes on the contact interface could hinder the direct contact between the ball and disk, leading to suppression of wear progression [34]. Particularly, this behavior of Ti_3_C_2_ was expected to be more significant at the edges of the contact area, as discussed earlier. It can also be seen that Ti_3_C_2_ was relatively more scattered on both the ball and disk surfaces with 5 wt% concentration (Figure 6c) than for 1 wt% concentration (Figure 6b). This may be responsible for more significant wear reduction as Ti_3_C_2_ concentration increased up to 5 wt%. However, it can be seen from Figure 6d that Ti_3_C_2_ was more agglomerated after the experiment for 7 wt% Ti_3_C_2_ concentration. Such agglomeration can be clearly observed from the SEM images of Ti_3_C_2_ on the ball surface after the experiment with various Ti_3_C_2_ concentrations, shown in Figure 8. It can be seen from the figure that the degree of agglomeration was significantly greater at 7 wt% Ti_3_C_2_ concentration than at 1 wt%–5 wt% Ti_3_C_2_ concentrations. It is plausible that more significant agglomeration of Ti_3_C_2_ at its high concentration may have been responsible for the reduction of the wear suppression effect, as proposed in a previous study [42]. Figure 9 shows SEM images of Ti_3_C_2_ and a tribo-film formed on the ball surface after experiments with 7 wt% Ti_3_C_2_ concentration under a 10 N normal force. The data in Figure 9 suggest damage of Ti_3_C_2_ and the formation of a tribo-film under relatively high normal force, which may contribute to suppressing the wear progression, as discussed earlier. It should further be noted that Ti_3_C_2_ can readily be oxidized in water, especially from the edges of the flakes, and TiO_2_ can be formed [47,48]. Hence, as a result of oxidation, the layered structure of Ti_3_C_2_ could be degraded, which may lead to a further decrease in its performance as an additive. However, such a performance decrease could be partly compensated, given that TiO_2_ may be used as a lubricant additive [17]. 

It should be noted that the tribological characteristics may vary significantly, depending on the material pair, and operating and environmental conditions. Hence, studies across a broad spectrum of experimental parameters are needed to elucidate the feasibility of Ti_3_C_2_ as an additive for water-based lubrication. In particular, a database to determine the optimal concentration should be established, given that optimal concentrations of lubricant additives may be dependent on the tribological system. Furthermore, the effects of size, thickness, and functional group on the tribological performance of Ti_3_C_2_ should be elucidated. In addition, the influence of dispersibility of Ti_3_C_2_ on the tribological characteristics should be explored to improve the performance of water-based lubrication. Long-term tests are further needed for the practical applicability of Ti_3_C_2_ as a lubricant additive. Nevertheless, the results of this work provide useful information for the fundamental tribological properties of Ti_3_C_2_ as a feasible water-based lubrication additive. 

## 4. Conclusions

In this work, the friction and wear characteristics of Ti_3_C_2_ as an additive for water-based lubrication were experimentally assessed using a ball-on-disk tribotester. The experiments were performed using SS balls and disks at varying normal forces and Ti_3_C_2_ concentrations. The overall results show that Ti_3_C_2_ can provide friction and wear reduction for water-based lubrication. The decrease in the direct contact between the ball and the disk offered by Ti_3_C_2_ flakes was believed to be responsible for this outcome. It was also shown that the hindering of contact was more significant at the edges of the contact interface, which in turn led to suppression of wear progression. Additionally, the degrees of reduction of friction and wear were found to increase from 7% to 20%, and from 20% to 48%, respectively, as Ti_3_C_2_ concentration increased from 1 wt% to 5 wt%, compared to the friction and wear without Ti_3_C_2_ additive. However, the reductions in friction and wear were limited to 10% and 23%, respectively, when Ti_3_C_2_ concentration further increased to 7 wt%. The excessive agglomeration of Ti_3_C_2_ flakes may have been responsible for this outcome. The results of this work may provide useful information for a fundamental understanding of the tribological properties of Ti_3_C_2_ as a water-based lubrication additive, and may therefore aid developing environment-friendly lubricants.

## References

[1] Panwar N.L., Kaushik S.C., Kothari S. (2011). Role of renewable energy sources in environmental protection: A review. Renew. Sustain. Energy Rev..

[2] Dornfeld D.A. (2014). Moving towards green and sustainable manufacturing. Int. J. Precis. Eng. Manuf. Green Technol..

[3] Więckowski W., Adamus J., Dyner M. (2020). Sheet metal forming using environmentally benign lubricant. Arch. Civ. Mech. Eng..

[4] Nosonovsky M., Bhushan B. (2010). Green tribology: Principles, research areas and challenges. Philos. Trans. A Math. Phys. Eng. Sci..

[5] Zhang S.-W. (2013). Green tribology: Fundamentals and future development. Friction.

[6] Anand A., Irfan Ul Haq M., Vohra K., Raina A., Wani M.F. (2017). Role of Green Tribology in Sustainability of Mechanical Systems: A State of the Art Survey. Mater. Today Proc..

[7] de Kraker A., van Ostayen R.A.J., Rixen D.J. (2007). Calculation of Stribeck curves for (water) lubricated journal bearings. Tribol. Int..

[8] Gao G., Yin Z., Jiang D., Zhang X. (2014). Numerical analysis of plain journal bearing under hydrodynamic lubrication by water. Tribol. Int..

[9] Ohana T., Suzuki M., Nakamura T., Tanaka A., Koga Y. (2006). Low-friction behaviour of diamond-like carbon films in a water environment. Diam. Relat. Mater..

[10] Wang Q., Zhou F., Wang X., Chen K., Wang M., Qian T., Li Y. (2011). Comparison of tribological properties of CrN, TiCN and TiAlN coatings sliding against SiC balls in water. Appl. Surf. Sci..

[11] Ding Q., Wang L., Wang Y., Wang S.C., Hu L., Xue Q. (2010). Improved Tribological Behavior of DLC Films Under Water Lubrication by Surface Texturing. Tribol. Lett..

[12] Yamakiri H., Sasaki S., Kurita T., Kasashima N. (2011). Effects of laser surface texturing on friction behavior of silicon nitride under lubrication with water. Tribol. Int..

[13] Chen C.-Y., Wu B.-H., Chung C.-J., Li W.-L., Chien C.-W., Wu P.-H., Cheng C.-W. (2013). Low-Friction Characteristics of Nanostructured Surfaces on Silicon Carbide for Water-Lubricated Seals. Tribol. Lett..

[14] Tomala A., Karpinska A., Werner W.S.M., Olver A., Störi H. (2010). Tribological properties of additives for water-based lubricants. Wear.

[15] Zhang C., Zhang S., Song S., Yang G., Yu L., Wu Z., Li X., Zhang P. (2014). Preparation and Tribological Properties of Surface-Capped Copper Nanoparticle as a Water-Based Lubricant Additive. Tribol. Lett..

[16] Alias A.A., Kinoshita H., Fujii M. (2015). Tribological properties of diamond nanoparticle additive in water under a lubrication between steel plate and tungsten carbide ball. J Adv. Mech. Des. Syst. Manuf..

[17] Wu H., Zhao J., Xia W., Cheng X., He A., Yun J.H., Wang L., Huang H., Jiao S., Huang L. (2017). A study of the tribological behaviour of TiO_2_ nano-additive water-based lubricants. Tribol. Int..

[18] Bao Y.Y., Sun J.L., Kong L.H. Tribological properties and lubricating mechanism of SiO_2_ nanoparticles in water-based fluid. Proceedings of the IOP Conference Series: Materials Science and Engineering, 17th IUMRS International Conference in Asia (IUMRS-ICA 2016).

[19] Jiang G., Yang Y. (2017). Preparation and tribology properties of water-soluble fullerene derivative nanoball. Arab. J. Chem..

[20] Peng Y., Hu Y., Wang H. (2006). Tribological behaviors of surfactant-functionalized carbon nanotubes as lubricant additive in water. Tribol. Lett..

[21] Berman D., Erdemir A., Sumant A.V. (2014). Graphene: A new emerging lubricant. Mater. Today.

[22] Kinoshita H., Nishina Y., Alias A.A., Fujii M. (2014). Tribological properties of monolayer graphene oxide sheets as water-based lubricant additives. Carbon.

[23] Xie H., Jiang B., Dai J., Peng C., Li C., Li Q., Pan F. (2018). Tribological Behaviors of Graphene and Graphene Oxide as Water-Based Lubricant Additives for Magnesium Alloy/Steel Contacts. Materials.

[24] Ky D.L.C., Khac B.C.T., Le C.T., Kim Y.S., Chung K.H. (2018). Friction characteristics of mechanically exfoliated and CVD-grown single-layer MoS_2_. Friction.

[25] Tran Khac B.C., DelRio F.W., Chung K.H. (2018). Interfacial Strength and Surface Damage Characteristics of Atomically Thin h-BN, MoS_2_, and Graphene. ACS Appl. Mater. Interfaces.

[26] Tran-Khac B.C., Kim H.J., DelRio F.W., Chung K.H. (2019). Operational and environmental conditions regulate the frictional behavior of two-dimensional materials. Appl. Surf. Sci..

[27] Cho D.-H., Kim J.-S., Kwon S.-H., Lee C., Lee Y.-Z. (2013). Evaluation of hexagonal boron nitride nano-sheets as a lubricant additive in water. Wear.

[28] Zhang B.M., Sun J.L. Tribological performances of multilayer-MoS_2_ nanoparticles in water-based lubricating fluid. Proceedings of the IOP Conference Series: Materials Science and Engineering, 17th IUMRS International Conference in Asia (IUMRS-ICA 2016).

[29] Anasori B., Lukatskaya M.R., Gogotsi Y. (2017). 2D metal carbides and nitrides (MXenes) for energy storage. Nat. Rev. Mater..

[30] Zhang D., Ashton M., Ostadhossein A., van Duin A.C.T., Hennig R.G., Sinnott S.B. (2017). Computational Study of Low Interlayer Friction in Ti_n+1_C_n_ (n = 1, 2, and 3) MXene. ACS Appl. Mater. Interfaces.

[31] Lian W., Mai Y., Liu C., Zhang L., Li S., Jie X. (2018). Two-dimensional Ti_3_C_2_ coating as an emerging protective solid-lubricant for tribology. Ceram. Int..

[32] Rosenkranz A., Grützmacher P.G., Espinoza R., Fuenzalida V.M., Blanco E., Escalona N., Gracia F.J., Villarroel R., Guo L., Kang R. (2019). Multi-layer Ti_3_C_2_T_x_-nanoparticles (MXenes) as solid lubricants—Role of surface terminations and intercalated water. Appl. Surf. Sci..

[33] Marian M., Tremmel S., Wartzack S., Song G., Wang B., Yu J., Rosenkranz A. (2020). Mxene nanosheets as an emerging solid lubricant for machine elements—Towards increased energy efficiency and service life. Appl. Surf. Sci..

[34] Liu Y., Zhang X., Dong S., Ye Z., Wei Y. (2016). Synthesis and tribological property of Ti_3_C_2_T_X_ nanosheets. J. Mater. Sci..

[35] Xue M., Wang Z., Yuan F., Zhang X., Wei W., Tang H., Li C. (2017). Preparation of TiO_2_/Ti_3_C_2_T_x_ hybrid nanocomposites and their tribological properties as base oil lubricant additives. RSC Adv..

[36] Yang J., Chen B., Song H., Tang H., Li C. (2014). Synthesis, characterization, and tribological properties of two-dimensional Ti_3_C_2_. Cryst. Res. Technol..

[37] Zhang X., Xue M., Yang X., Wang Z., Luo G., Huang Z., Sui X., Li C. (2015). Preparation and tribological properties of Ti_3_C_2_(OH)_2_ nanosheets as additives in base oil. RSC Adv..

[38] Chen J., Zhao W. (2020). Simple method for preparing nanometer thick Ti_3_C_2_T_X_ sheets towards highly efficient lubrication and wear resistance. Tribol. Int..

[39] Feng W., Luo H., Wang Y., Zeng S., Deng L., Zhou X., Zhang H., Peng S. (2018). Ti_3_C_2_ MXene: A promising microwave absorbing material. RSC Adv..

[40] Hertz H.R. (1882). Ueber die Berührung fester elastischer Körper. J. Die Reine Angew. Math. (Crelles J.).

[41] Blau P.J. (2005). On the nature of running-in. Tribol. Int..

[42] Guo Y.-B., Zhang S.-W. (2016). The Tribological Properties of Multi-Layered Graphene as Additives of PAO2 Oil in Steel–Steel Contacts. Lubricants.

[43] Wu P., Chen X., Zhang C., Zhang J., Luo J., Zhang J. (2020). Modified graphene as novel lubricating additive with high dispersion stability in oil. Friction.

[44] Çelik O.N., Ay N., Göncü Y. (2013). Effect of Nano Hexagonal Boron Nitride Lubricant Additives on the Friction and Wear Properties of AISI 4140 Steel. Part. Sci. Technol..

[45] Mousavi S.B., Heris S.Z., Estelle P. (2020). Experimental comparison between ZnO and MoS_2_ nanoparticles as additives on performance of diesel oil-based nano lubricant. Sci. Rep..

[46] Rabinowicz E. (1995). Friction and Wear of Materials.

[47] Zhang C.J., Pinilla S., McEvoy N., Cullen C.P., Anasori B., Long E., Park S.-H., Seral-Ascaso A., Shmeliov A., Krishnan D. (2017). Oxidation Stability of Colloidal Two-Dimensional Titanium Carbides (MXenes). Chem. Mater..

[48] Ghassemi H., Harlow W., Mashtalir O., Beidaghi M., Lukatskaya M.R., Gogotsi Y., Taheri M.L. (2014). In situ environmental transmission electron microscopy study of oxidation of two-dimensional Ti_3_C_2_ and formation of carbon-supported TiO_2_. J. Mater. Chem. A.

